# Subclinical and long-term effects of severe acute respiratory syndrome coronavirus 2 infection in Danish farmed mink: implications for disease surveillance

**DOI:** 10.1186/s13028-025-00813-w

**Published:** 2025-06-02

**Authors:** Michelle Lauge Quaade, Mia Mylin Jensen, Thomas Bruun Rasmussen, Tim Kåre Jensen, Anne Sofie Hammer

**Affiliations:** 1 Department of Veterinary and Animal Sciences, Faculty of Health and Medical Sciences, University of Copenhagen, Ridebanevej 3, 1870 Frederiksberg C, Denmark; 2 Department of Virus and Microbiological Special Diagnostics, Statens Serum Institut, Artillerivej 5, 2300 Copenhagen S, Denmark

**Keywords:** COVID-19, Histopathology, Immunohistochemistry, Mink, Pathology, SARS-CoV-2

## Abstract

**Background:**

The COVID-19 pandemic has caused over 776 million confirmed cases and more than 7 million deaths worldwide. In addition to humans, various animal species have exhibited natural infections, with mink being the only farmed animals consistently linked to severe illness and zoonotic transmission to humans. This study investigates histological pulmonary lesions in Danish farm mink infected with severe acute respiratory syndrome coronavirus 2 (SARS-CoV-2), focusing on groups with different clinical signs and outcomes.

**Results:**

Histopathological evaluations revealed lesions in SARS-CoV-2-positive mink with and without clinical signs of disease. The main findings in lungs from SARS-CoV-2-positive mink in all study groups were extensive respiratory epithelial damage, acute diffuse alveolar damage, and vascular lesions, including the formation of thrombi. Additionally, immunohistochemical staining confirmed the presence of viral particles primarily in the respiratory epithelia. Lymphoid cells exhibited nodular and perivascular aggregates similar to bronchus-associated lymphoid tissue in older SARS-CoV-2 infected and uninfected mink, indicating a potential age-related feature of mink lungs.

**Conclusions:**

The presence of subclinical and long-term pulmonary lesions associated with SARS-CoV-2 infections in farm mink suggests that the impact of outbreaks may be more serious than clinical signs records indicate. The current SARS-CoV-2 surveillance system on Danish mink farms does not properly address such problems and repeated outbreaks on farms could occur without detection if there are no clinical signs or increased mortality due to SARS-CoV-2. The severity of subclinical lesions reveals hidden health and welfare challenges in mink, underscoring the need for improved prevention measures, surveillance and understanding of long-term impact of SARS-CoV-2 infection in mink.

**Supplementary Information:**

The online version contains supplementary material available at 10.1186/s13028-025-00813-w.

## Background

As of November 2024, the coronavirus disease 2019 (COVID-19) pandemic has produced more than 776 million confirmed human cases and more than 7 million deaths worldwide according to the World Health Organization [1]. Additionally, cases of natural infections have been reported in a wide range of animals including dogs, cats, otters, zoo felids, zoo gorillas, ferrets, and mink [2–7].

Shortly after the COVID-19 pandemic in humans began in early 2020, the first outbreaks of severe acute respiratory syndrome coronavirus 2 (SARS-CoV-2) on domestic mink farms were identified in the Netherlands in April 2020 [8]. These were followed by outbreaks on Danish mink farms from June 2020 [9, 10]. Thereafter, SARS-CoV-2 infections were reported on mink farms in many countries including the United States, Spain, Sweden, Italy, France, Greece, Lithuania, Canada, Poland, and Latvia [5, 11–16]. Danish mink production faced a significant challenge with outbreaks recorded on 290 mink farms by November 2020. The Danish Government subsequently authorized culling on all mink farms on 4 November 2020 [9]. Prior to the culling, Denmark was a leading contributor to the global mink fur industry with more than 1,000 privately owned farms and approximately 2.5 million breeding mink producing around 12.5 million pelts annually [17]. As of November 2024, there are five mink farms with approximately 35,000 mink in total registered in Denmark [18] and a SARS-CoV-2 surveillance program has been initiated. This is based on collection of throat swabs to test for SARS-CoV-2 at various intervals during the year, as well as monitoring for clinical signs [19].

Reports of observations on SARS-CoV-2 infected mink farms range from mostly asymptomatic in the Danish and Spanish outbreaks [16, 20] to various clinical signs, including watery nasal discharge, respiratory distress, anorexia, mild depression, and coughing in the Dutch, Greek, and American outbreaks [8, 11, 21, 22]. Mortality rates range from minimal (0.45%) in Denmark [20], 2.4% in the Netherlands [8, 22], 8–10% in Greece [21], and up to 55% in the United States [11].

While few cases of zoonotic transmission from white-tailed deer [23] and hamsters [24] have been reported, farmed mink are the species with the most widespread confirmed cases of animal-to-human transmission [10, 25]. They are the only nonhuman species consistently associated with severe illness and death [8, 11, 21, 22] and are the second most commonly documented species exhibiting SARS-CoV-2 infections [26]. However, data regarding the pathology of SARS-CoV-2 infection in mink remain limited. To date, descriptions of SARS-CoV-2 outbreaks in domestic mink in the Netherlands, Greece, Spain, and the USA have reported histopathological similarities to the lung tissue of human COVID-19 patients [11, 16, 21, 22, 27].

This study investigates pulmonary lesions in Danish farm mink (*Neogale vison*) naturally infected with SARS-CoV-2. We studied a group of asymptomatic mink kits, a group of mink kits that had severe respiratory disease with fatal outcome, and a group of mink kits that were in recovery from infection. Two groups of healthy control mink, matched by age to the three case groups, were also included. All the mink were from farms that were free from Aleutian disease (AD).

## Methods

### Study design and animals

We investigated samples of lung tissue collected from mink infected with SARS-CoV-2 and uninfected control mink. The investigation was designed as an observational case–control study. The study included three case groups and two control groups, as summarized in Table 1. Case groups were matched with control groups of a similar age from farms with no history of SARS-CoV-2 outbreaks. Additionally, control animals were confirmed negative for active and previous SARS-CoV-2 infection by reverse transcription-quantitative polymerase chain reaction (RT-qPCR) on throat swabs and antibody enzyme-linked immunosorbent assay (ELISA) on blood samples or by SARS-CoV nucleocapsid immunohistochemistry and viral metagenomics. These results are summarized in Table 2. Animals from the subclinical SARS-CoV-2 (SCS), controls July (Con_July), controls November (Con_Nov), and recovering SARS-CoV-2 (RS) groups were randomly selected by the farmer. Animals in the SCS were a subset of the animals sampled on 22 June 2020, in a previous study [10]. Animals in the RS group were a subset of animals from a previously described study, collected on 5 October 2020 [28]. Animals in the SCS, RS, and control groups were euthanized after sedation, in accordance with protocols previously described [29]. All animals in the clinical SARS-CoV-2 (CS) group were found dead on the farm and collected within a few hours postmortem. All animals were stored overnight at 5 °C and necropsies were performed the following day.

**Table 1:** Summary of study groups

Group	Group name	Date, sampling	Sampling	Description
Subclinical SARS-CoV-2 *(n* = *14)*	SCS	June 22, 2020	Random, euthanized	Asymptomatic SARS-CoV-2 infected mink kits from a farm with infected personnel [10]. 2 months old
Controls, July *(n* = *10)*	Con_July	July 2017	Random, euthanized	Healthy mink kits from a farm with no history of SARS-CoV-2 infection. 2 months old
Clinical SARS-CoV-2 *(n* = *13)*	CS	Oct 5, 2020	All dead animals on sampling date	SARS-CoV-2 infected mink kits from an outbreak, exhibiting severe respiratory distress and fatalities. 5 months old
Controls, November *(n* = *30)*	Con_Nov	Nov 4, 2020	Random, euthanized	Healthy mink kits from a farm with no history of SARS-CoV-2 infection. 6 months old
Recovering SARS-CoV-2* (n* = *10)*	RS	Oct 5, 2020	Random, euthanized	Mink kits recovering from SARS-CoV-2 infection, collected one month after clinical signs ceased; included reduced feed intake and a few cases of diarrhea and respiratory signs [28]. 5 months old

*SARS-CoV-2* severe acute respiratory syndrome coronavirus 2

**Table 2:** Results of three SARS-CoV-2 detection methods in all study groups

Group	*n*	SARS-CoV-2 RT-q*PCR*	SARS-CoV-2 antibody ELISA	SARS-CoV nucleocapsid IHC
		*(positive/tested)*	*(positive/tested)*	*(positive/tested)*
SCS	*14*	11/14	1/14	9/14
Con_July	*9*	–*	–	0/9
CS	*13*	13/13	–	5/5
Con_Nov	*30*	0/30	0/30	0/5
RS	*10*	0/10	10/10	0/10

Results from different viral detection methods including SARS-CoV-2 antigen in throat swabs using RT-qPCR, anti-SARS-CoV-2 antibodies in blood samples using ELISA, and SARS-CoV nucleocapsid IHC in lung tissue for the five study groups of SARS-CoV-2 infected and uninfected mink kits

*SARS‑CoV‑2* severe acute respiratory syndrome coronavirus 2, *ELISA* enzyme-linked immunosorbent assay, *IHC* immunohistochemistry, *SCS* subclinical SARS-CoV-2, *Con_July* controls from July, *CS* clinical SARS-CoV-2, *Con_Nov* controls from November, *RS* recovering SARS-CoV-2, *RT-qPCR* reverse transcription-quantitative polymerase chain reaction

^*^Samples from Con_July animals were analyzed using viral metagenomics and no coronavirus was detected

Cardiac blood samples were collected from all animals in the SCS, Con_Nov, and RS groups at the time of euthanasia. Throat swabs were also obtained from all animals in the SCS, CS, Con_Nov, and RS groups. No blood samples were collected from animals in the CS group.

No ethical or laboratory animal permits were required according to Danish legislation. All included animals were destined for euthanasia, due to annual pelting or in accordance with national legislation for disease control.

### Necropsy

Standard necropsies were conducted as previously described [30]. The body condition of each carcass was scored on a scale of 1–5, in accordance with relevant guidelines [31]. For each animal, sex, age (juvenile/adult), and gross pathological findings were also recorded.

### Histopathology

Samples from various organs were collected during necropsies, including cranial and caudal lung lobes from each mink. Tissue samples were fixed in 10% neutral buffered formalin for 2 d. After trimming, tissues from the cranial and caudal lung lobes were processed routinely for histology: Dehydrated using a graded series of ethanol washes (75%, 80%, 96%, and 99%), then treated with xylene and embedded in paraffin. Next, tissue sections (2–4 µm) were stained with hematoxylin and eosin. Fibrin was visualized using Martius scarlet blue stain, following a modified protocol previously described [32]. Collagen was visualized with Masson’s trichrome stain using an adapted technique detailed earlier [33].

A semiquantitative scoring protocol for pulmonary lesions was developed. In this scoring system, the following six main categories were divided into subcategories: (1) bronchi, (2) glands, (3) bronchioles, (4) vascular system, 95) interstitium, and (6) alveolar lumen. For the bronchi and bronchioles categories, the subcategories included scoring of epithelial damage, luminal secretion and hemorrhage, and submucosal inflammation. For the glands category, the subcategories included scoring of cellular inflammation and degeneration. For the vascular system category, the subcategories included scoring of vasculitis, perivasculitis, perivascular edema, and perivascular accumulation of mononuclear cells. For the interstitium category, the subcategories included scoring of cell infiltration, type II pneumocyte proliferation, and alveolar damage. For the alveolar lumen category, the subcategories included scoring of inflammation, alveolar fibrin, luminal edema, and an overall evaluation of reduction of airspace (for detailed descriptions, see Additional file 1). Each animal was assigned a combined score for each main category. This combined score was the sum of the scores from each of the relevant subcategories. Scores were assigned on an ordinal scale from 0 to 3, with 0 indicating no pathological lesions, and scores of 1 to 3 indicating increasing severity of pathological lesions (a score of 3 indicates the most severe lesions).

Additionally, a dichotomous scale (present/not present) was used to evaluate the presence or absence of the following: (1) hemosiderin in macrophages; (2) giant cells; (3) bacteria; (4) thrombi; (5) neutrophils in lumen or septae; and (6) pulmonary corpora amylacea (PCA).

All tissue sections were single blinded prior to evaluation. The evaluation was performed by the same person in the two case–control groups and the evaluation process was supervised by the same senior pathologists throughout. A Leica DM4 B microscope was used for reading and images were obtained using a Leica DFC7000 T camera and Leica Application Suite X software, version 3.7.6.25997 (Leica Microsystems, Wetzlar, Germany).

### Immunohistochemistry

Immunohistochemistry (IHC) markers applied in this study included the following: (1) MAC374G/MAC287 IHC antibody (Bio Rad Laboratories A/S, Copenhagen, Denmark) for identifying macrophages; (2) SARS-CoV nucleocapsid IHC antibody (Nordic Biosite ApS, Copenhagen, Denmark) for identifying SARS-CoV-2 infected cells; and 3) CD3 and CD79 stains (both DAKO Denmark ApS, Glostrup, Denmark) for identifying T and B lymphocytes, respectively. The MAC387, CD3, and CD79 IHC procedures were used on all tissue sections from the CS and Con_Nov groups. The SARS-CoV nucleocapsid IHC procedure was used on tissue samples from the Con_July, SCS, and RS groups, and on five randomly selected tissue sections from the Con_Nov and CS groups. The interpretation of IHC results was based on the presence, localization, and intensity of staining. Positive staining was defined as specific chromogenic signals localized within the expected cellular compartments (e.g., cytoplasmic or nuclear) compared to negative controls. The absence of staining or nonspecific background signal was recorded as negative.

### Virology

RT-qPCR, serology evaluation, and sequencing were performed as previously described [10]. Throat swabs were analyzed for SARS-CoV-2 viral RNA by RT-qPCR, in accordance with the procedure described by Corman et al*.* [34]. Blood samples were analyzed for the presence of anti-SARS-CoV-2 antibodies using ELISA (Beijing Wantai Biological Pharmacy Enterprise, Beijing, China), as previously described [10]. Sequencing of individual variants and determination of relationship to the pangolin lineage were performed as previously described [28, 35]. All included farms were confirmed free of antibodies specific for Aleutian mink disease virus (AMDV).

### Statistics

For all data, non-parametric tests were employed, as no assumptions of normal distribution were made.

Wilcoxon’s signed-rank test was used to investigate any differences between samples from the cranial and caudal lung lobes in the same individual from the Con_Nov and CS groups; the Mann–Whitney U test was used to investigate differences between scores from the same groups. The Kruskal–Wallis test with Dunn’s correction for multiple comparisons was used to evaluate the sum of scores across all groups included in the study. A P-value < 0.05 was considered statistically significant. All statistical analyses were performed using GraphPad Prism software (ver. 10.0; GraphPad Software, San Diego, CA, USA). When tissue samples were randomly selected for staining, the RAND()-function in Excel software (2016; Microsoft Corporation, Redmond, WA, USA) was used.

## Results

### Gross pathology

All CS mink kits had enlarged dark-red lungs with increased texture. No other gross lesions were recorded.

In the SCS group, five animals had congested lungs with increased texture and nine animals had no apparent gross lesions.

One animal from the Con_Nov control group had congested lungs. In the RS group, all animals had an enlarged congested spleen and mild steatosis and only one animal did not have lungs with increased texture.

### Histopathology

**Fig. 1:**
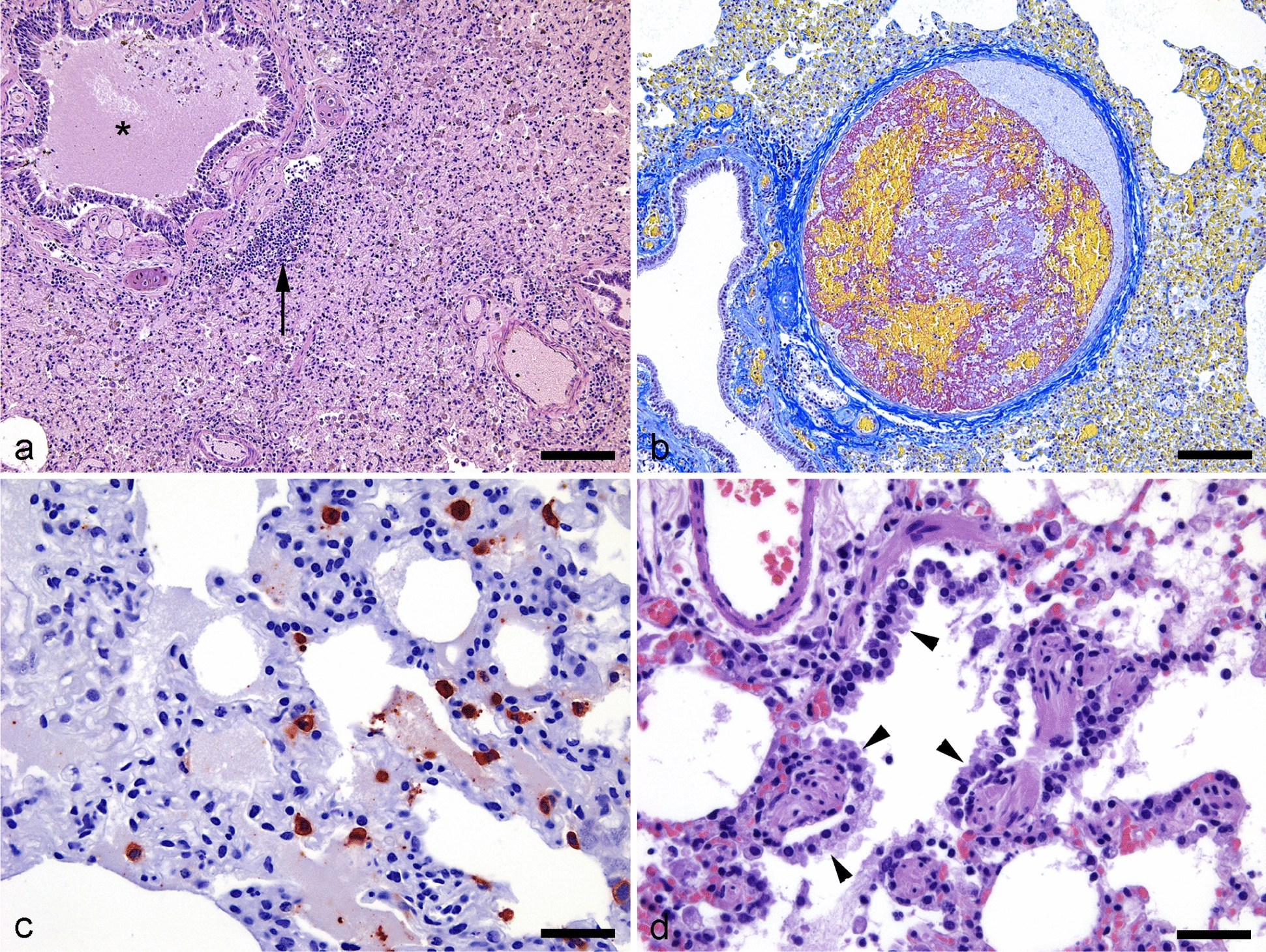
Histological images of SARS-CoV-2 infected and recovering mink. Histological findings in SARS-CoV-2 infected mink with severe clinical disease (**a**,** b**,** c**) and in recovery (**d**). **a** Severe luminal secretion in a bronchi (asterix), submucosal inflammation (arrow), and severe diffuse alveolar damage (HE, bar = 100 µm). **b** Acute heterogeneous fibrin thrombi in a pulmonary artery consisting of organized fibrin (red) and erythrocytes (yellow) (MSB, bar = 100 µm). **c** SARS-CoV nucleocapsid positive cells in the alveolar lumen and wall (red) (SARS-CoV nucleocapsid IHC, bar = 25 µm). **d** Type II pneumocyte proliferation (arrowheads) in (HE, bar = 25 µm). *SARS-CoV-2* severe acute respiratory syndrome coronavirus 2, *HE* hematoxylin and eosin, *MSB* martius scarlet blue

Histopathological examination of lung tissue samples from all animals in the SCS, CS, and RS groups was used to identify lesions; tissue samples from the CS group exhibited the most severe lesions. Representative histological images from the Con_July group and each case group (SCS, CS, and RS) are shown in Fig. 1.

**Fig. 2:**
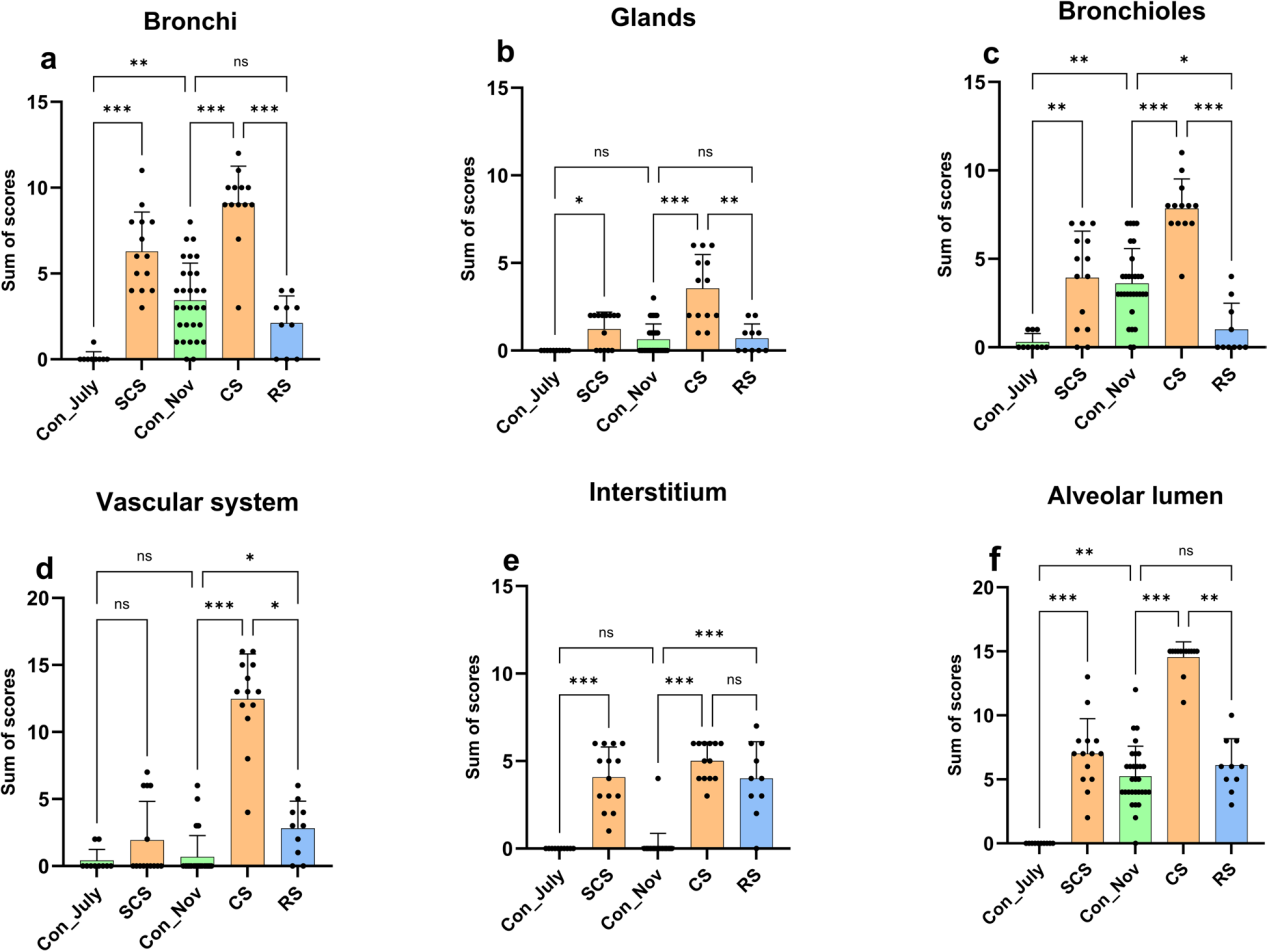
Histopathological scores for six main categories assigned to SARS-CoV-2 infected, uninfected, and recovering mink. Scatter plots with bars showing the mean and standard deviation of the sum of scores assigned to each group: Con_July (*n* = 9), SCS (*n* = 14), Con_Nov (*n* = 30), CS (*n* = 13), and RS (*n* = 10). For each category, a semiquantitative scoring protocol was used: **a** bronchi (epithelial damage, luminal secretion and hemorrhage, and submucosal inflammation); **b** glands (inflammation and degeneration); **c** bronchioles (epithelial damage, luminal secretion and hemorrhage, and submucosal inflammation); **d** vascular system (vasculitis, perivasculitis, perivascular edema, and perivascular accumulation of mononuclear cells); **e** interstitium (cell infiltration, type II pneumocyte proliferation, and alveolar damage); and **f** alveolar lumen (inflammation, fibrin, hemorrhage, edema, and overall reduction of airspace). Statistical analysis was performed using the Kruskal–Wallis test and Dunn’s correction for multiple comparisons. A *P*-value < 0.05 was considered statistically significant (ns ≥ 0.05, * < 0.05, ** < 0.01,*** < 0.001). *ns* not significant, *SARS‑CoV‑2* severe acute respiratory syndrome coronavirus 2, *Con_July* healthy controls from July, *SCS* subclinical SARS-CoV-2, *Con_Nov* healthy controls from November, *CS* clinical SARS-CoV-2, *RS* recovering from SARS-CoV-2

Regarding the scores, the CS group exhibited significantly higher scores across all main categories compared to the corresponding Con_Nov group. A similar trend was observed in the SCS group when compared to Con_July group; however, there was no significant difference between these two groups for the"vascular system"category. The RS group scores were significantly lower than the CS group scores for all categories except the"interstitium"category, where there was no significant difference. The RS group had significantly higher scores than the Con_Nov group for the"vascular system"and"interstitium"categories but significantly lower scores than the Con_Nov group for the"bronchioles"category. The Con_July group had significantly lower scores than the Con_Nov group for the “bronchi,” “bronchioles,” and “alveolar lumen” categories, but there were no significant differences between these groups for the remaining categories (Fig. 2). One control mink from the Con_July group was excluded due to the presence of histological lesions that indicated bronchopneumonia associated with bacterial infection.

**Fig. 3:**
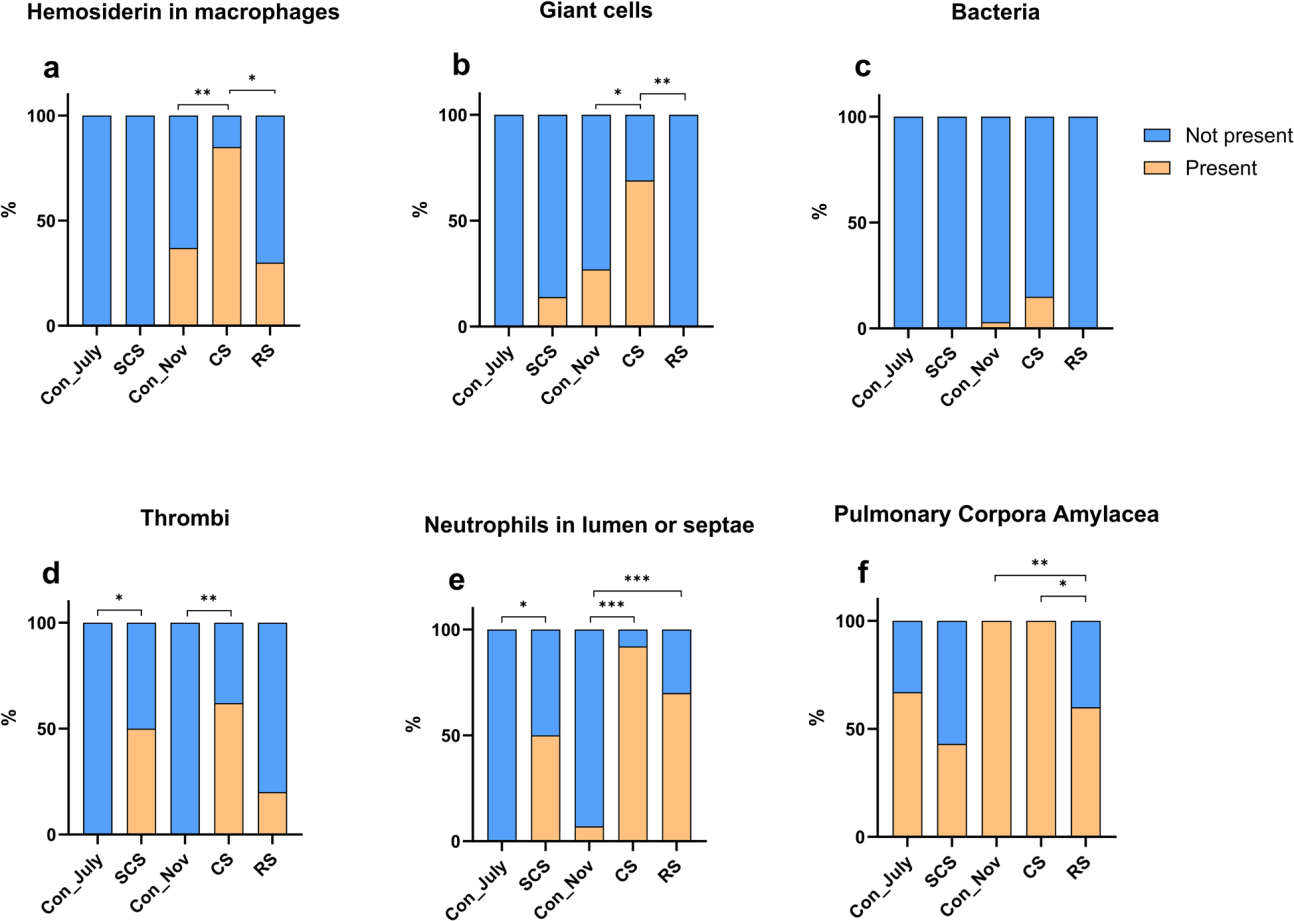
Histological characteristics of SARS-CoV-2 infected, uninfected, and recovering mink. Stacked bar chart showing the histological characteristics of the five study groups: Con_July (*n* = 9), SCS (*n* = 14), Con_Nov (*n* = 30), CS (*n* = 13), and RS (*n* = 10). Each graph represents a particular characteristic including: **a** hemosiderin in macrophages, **b** giant cells, **c** bacteria, **d** thrombi, **e** neutrophils in lumen or septae, and **f** pulmonary corpora amylacea. Each bar corresponds to a group, with segments indicating the proportions that are “present” (represented in orange) and “not present” (represented in blue). Fisher’s exact test was used to evaluate differences in outcomes between the groups. A *P*-value < 0.05 was considered statistically significant (ns ≥ 0.05, * < 0.05, ** < 0.01, *** < 0.001). *ns* not significant, *SARS‑CoV‑2* severe acute respiratory syndrome coronavirus 2, *Con_July* controls from July, *SCS* subclinical SARS-CoV-2, *Con_Nov* controls from November, *CS* clinical SARS-CoV-2, *RS*: recovering from SARS-CoV-2

There was significantly more intracellular hemosiderin in macrophages from the CS group (11/13; 85%) than in those from the Con_Nov (11/30; 37%) and RS (3/10; 30%) groups, whereas no intracellular hemosiderin was observed in macrophages from either the SCS or Con_July group. Giant cells were observed in the SCS (2/14; 14%), Con_Nov (8/30; 27%), and CS (9/13; 69%) groups, with significantly more being present in the CS group than in the Con_Nov group. Bacteria, mainly cocci, were only observed in three cases, with no significant differences between the Con_Nov (1/30; 3%) and CS (2/13; 15%) groups. Thrombi were observed in the SCS (7/14; 50%), CS (8/13; 62%), and RS (2/10; 20%; Fig. 1) groups but not in the corresponding control groups (Con_July and Con_Nov). There was no significant difference in thrombi formation between the CS and RS groups. There were significantly more neutrophils in the alveolar lumen or septae of the SCS (7/14; 50%), CS (12/13; 92%), and RS (6/10; 60%) groups than in the corresponding Con_July (0%) and Con_Nov (2/30; 7%) control groups. Finally, there were round, solitary, acellular and eosinophilic structures in the alveolar lumen that resembled PCA in all groups, but these were significantly more abundant in the CS and Con_Nov groups than in the RS group. Significant differences between the groups are shown in Fig. 3.

In addition to the scores obtained from the semiquantitative protocol, there were notable differences in the particular histological lesions present in the different groups, including vasculitis, perivasculitis, cell infiltration in the alveolar interstitium, and type II pneumocyte proliferation. For example, mild (9/13; 69%) to moderate (3/13; 23%) vasculitis was exclusively observed in the CS group.

Perivasculitis was only observed in the CS group, with all animals displaying some degree of perivasculitis (mild, (1/13;8%); moderate, (2/13;15%); or severe, (10/13;77%)). All groups exhibited perivascular accumulation of mononuclear cells, especially the RS group. Varying degrees of perivascular edema were observed in the SCS and CS groups.

Cell infiltration of mononuclear cells or neutrophil granulocytes in the alveolar interstitium was observed in the SCS, CS, and RS groups, with 12 out of 13 (92%) of the tissue sections in the CS group showing a diffuse pattern of cell infiltration. The infiltration patterns in the SCS and RS groups were milder, including focal (4/14 (29%) and 1/10 (10%), respectively), multifocal (3/14 (21%) and 2/10 (20%), respectively), and diffuse (5/14 (36%) and 3/10 (30%), respectively) patterns.

Type II pneumocyte proliferation was infrequent and included focal (3/14 (21%) in SCS and 2/10 (20%) in RS) and multifocal (1/14 (7%) in SCS and 4/10 (40%) in RS) proliferation. No proliferation was observed in the CS or control groups. Diffuse alveolar damage was observed to varying degrees in the SCS, CS, and RS groups.

**Fig. 4:**
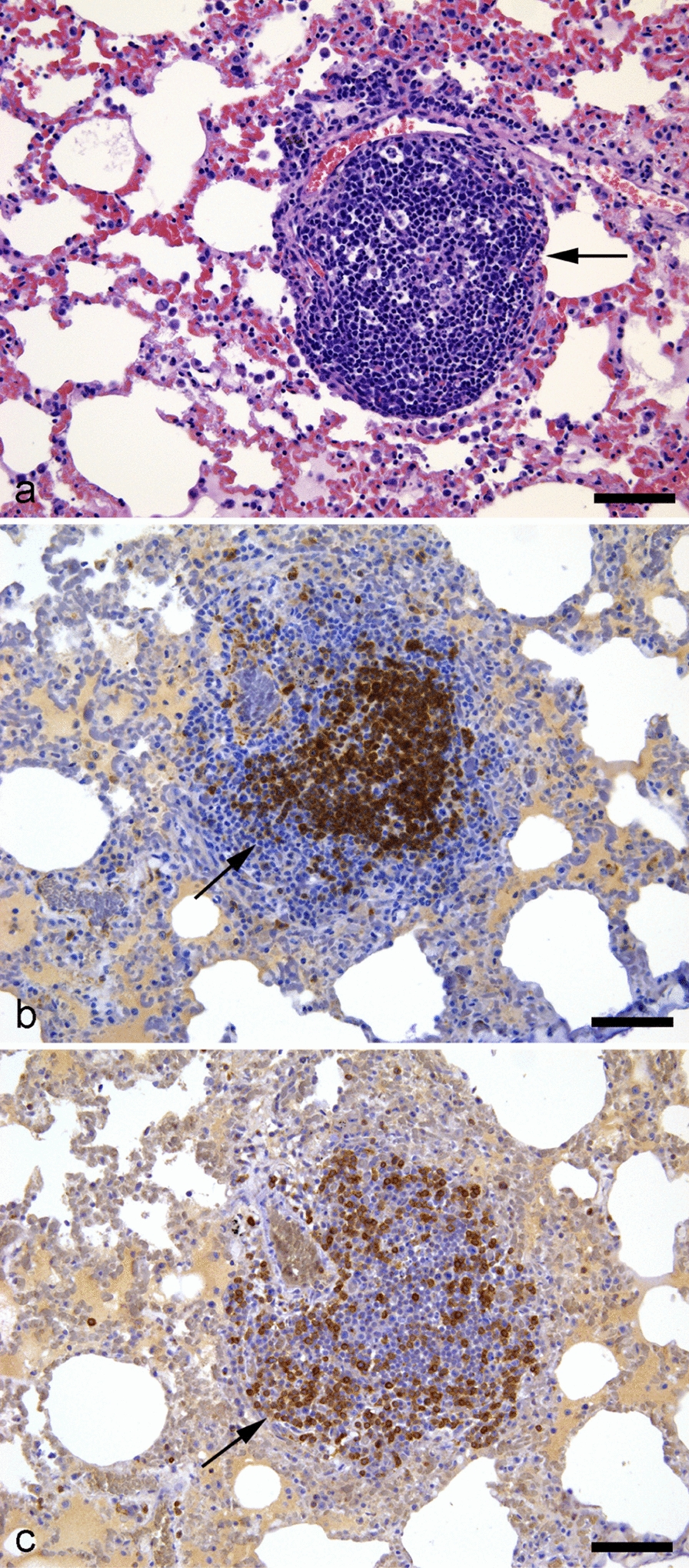
Secondary lymphoid tissue in an apparently healthy mink. Lung tissue from an apparently healthy mink, showing aggregates of lymphocytes (**a**), predominantly B cells (**b**), and T cells (**c**) in the surrounding regions (all indicated by arrows). **a** hematoxylin and eosin; **b** CD79 stain; **c** CD3 stain. Scale bars: 50 µm for all images

In both the Con_Nov and RS groups, there was multifocal nodular accumulation of mononuclear cells at several locations. Most frequently, the nodules were peribronchiolar and associated with bifurcations; however, in 8 out of 30 animals from the Con_Nov group (27%) and 6 out of 10 animals from the RS group (60%), focal mononuclear nodules were present in the interstitia of the alveolar tissue (Fig. 4).

Cranial and caudal lung sections from the CS and Con_Nov groups were evaluated. Upon unblinding, the scores for the cranial and caudal lung lobes of each animal were compared. Because there were no statistically significant differences between these scores, each animal was assigned the higher score from the two lobes in each subcategory.

All lung sections from the RS group and six randomly selected sections from the Con_Nov group were stained with Masson’s trichrome to evaluate lung fibrosis. We observed no differences in fibrosis between the two groups.

### Immunohistochemistry

The MAC387 IHC evaluation revealed macrophages in lung tissue from both groups. In the CS group, macrophages infiltrated the vascular wall in cases of vasculitis and often when interstitial inflammation was observed. In the Con_Nov group, there were macrophages at various locations within the lung. Specifically, macrophages were found multifocally infiltrating the alveolar interstitium (up to two cells per alveolar septum), in small numbers in the lumina of the bronchi and bronchioles (and sporadically subepithelially), and in *lamina propria* of both bronchi and bronchioles.

In both the SCS and CS groups, SARS-CoV nucleocapsid positive cells were observed in the bronchi and bronchiolar epithelium, in the alveolar wall, and in the alveolar lumen. In general, the concentration of SARS-CoV nucleocapsid positive pneumocytes and macrophages was higher near the bronchi and bronchioles than in the peripheral pulmonary tissue. SARS-CoV nucleocapsid positive epithelial cells in the bronchi and bronchioles retained normal morphology with intact cilia in the pseudostratified epithelium and were attached to the basement membrane. SARS-CoV nucleocapsid positive pneumocytes were either located in the alveolar wall or desquamated to the alveolar lumen. SARS-CoV nucleocapsid positive mononuclear cells were found in the lumen of alveoli, bronchi and bronchioles, and in the alveolar interstitium. MAC387 staining indicated that the positive cells in the alveolar lumen were probably macrophages. In three samples from the CS group, glandular cells were SARS-CoV nucleocapsid positive. No endothelia in any vessels or leukocytes involved in vasculitis were positive. No positive cells were associated with thrombi formation. No SARS-CoV nucleocapsid positive cells were observed in the Con_July, Con_Nov, or RS group.

CD3 and CD79 IHC analysis revealed that nodular accumulations of mononuclear cells observed in alveolar interstitia in the Con_Nov group consisted of B lymphocytes at the center and T lymphocytes at the periphery and resembled secondary lymphoid tissue. There was perivascular lymphocytic cuffing in both the Con_Nov and CS groups, predominantly involving T lymphocytes. In the CS group, however, this perivascular cuffing consisted of several layers of T lymphocytes.

### Virology

A summary of the results from three different SARS-CoV-2 detection methods including SARS-CoV-2 antigen in throat swabs using RT-qPCR, anti-SARS-CoV-2 antibodies in blood samples using ELISA, and SARS-CoV nucleocapsid IHC on pulmonary histology sections is shown in Table 2.

## Discussion

Overall, the histopathological evaluation in this study revealed extensive pulmonary pathology in all case groups. The pathological findings were significantly more severe in the group of mink that developed clinical signs of severe acute respiratory distress and died due to SARS-CoV-2 infection. However, noticeable lesions were also observed in the group of mink that exhibited no clinical disease, as well as in those that were in recovery from a SARS-CoV-2 outbreak. In a previous study, mild to moderate bronchointerstitial pneumonia was reported in asymptomatic SARS-CoV-2-infected mink during an outbreak on a Spanish farm [16]. Additionally, another study found that one in three mink farms with SARS-CoV-2 infections showed no clinical signs of disease [20].

The mandatory SARS-CoV-2 surveillance program in Denmark, as of 2025, focuses on detecting infections linked to clinical disease in mink farms. Farms are subject to public monitoring if there are any suspicions of SARS-CoV-2 infection based on observed clinical signs. Additionally. the program mandates the collection of throat swabs to test for virus from 5 deceased animals every 3 months, from 15 animals no more than 10 days before pelting, and from 60 animals prior to import to Denmark when new animals are purchased abroad [19].

Originally native to the North American continent, the mink is a solitary carnivore with territorial, crepuscular, and nocturnal habits [36]. Its inclination toward solitude and masking signs of pain mean that assessing disease symptoms in mink may be difficult, similar to other domestic carnivores such as cats [37] and ferrets [38]. Good health and freedom from injury are welfare quality principles that have been included in the WelFur assessment program, which has been implemented at mink farms across the European continent and is maintained by impartial assessors. In this context, good health is defined by the following welfare quality criteria: absence of injuries, skin lesions or other injuries to the body; freedom from disease, risk of mortality, diarrhea, lameness, impaired movement, or pain produced as a result of management procedures [39]. A previous study in the Netherlands suggested that outbreaks of SARS-CoV-2 on some mink farms may be missed due to a lack of clinical signs [22]. In this study, the severity of pathology observed in asymptomatic SARS-CoV-2-infected mink, as well as those that were in recovery from the disease, suggests that a surveillance strategy based on testing four times a year and monitoring clinical signs may not adequately meet welfare needs. First, infected mink with subclinical disease may never be tested under the current surveillance system; unless they die from other diseases, they will not be included in the mandatory testing every three months. Second, testing deceased mink with throat swabs every three months appears insufficient, as they may test negative just one month after the initial detection of an outbreak, as previously reported [28]. Danish animal welfare legislation requires that individuals responsible for production animal care monitor each animal at least once daily. While mink farmers routinely observe their animals as part of standard management practices, systematic studies on the presentation of clinical diseases, including signs of pain-related behaviors, have not been reported in mink, unlike other more commonly studied animal models or production animals. This highlights the need for further research to establish reliable behavioral and physiological markers of disease and discomfort in this species. Consequently, SARS-CoV-2 infection on a farm could be overlooked in the present surveillance program. Furthermore, the public health implications of undetected SARS-CoV-2 infection on mink farms should be investigated further. The evidence of the mink-adapted SARS-CoV-2 Cluster 5 variant spilling back into the local community [8, 10, 40] underlines a need for improved biosecurity measures on mink farms in general. Although the mutations in the Cluster 5 variant negatively impacted the replication of the virus in human cells [41] the spread of this variant into the human community underscores the possibility of mink serving as reservoirs of new virus variants with unknown pathogenicity in the future.

Additionally, perivasculitis was frequently observed in the mink group that succumbed to SARS-CoV-2 infection, which corroborates reports from previous studies on mink [11, 16, 22, 42]. Notably, perivascular accumulations consisting of multiple layers of mononuclear cells were also consistently present in the control groups. A previous study found that mononuclear vascular cuffing was common in Danish mink kits, but no infectious cause was identified [43]. This may be similar to the perivascular lymphocytic cuffing documented in clinically unremarkable domestic cats [44]. Consequently, the term “perivasculitis” may be misleading and apparent lesions in lung tissues from farmed mink could represent the proliferation of pre-existing perivascular lymphatic tissue rather than true perivasculitis. Additionally, nodular structures resembling secondary lymphoid tissue, featuring a central B follicle and an outer T-lymphocyte zone, were consistently observed in the control group from November and in the group of mink in recovery from SARS-CoV-2, in which the kits were 6 months and 5 months old, respectively. These lymphoid aggregates share features with the bronchus-associated lymphoid tissue (BALT) described in other species, including cats and humans [45–47]. Additionally, such lymphoid aggregates were not present in younger mink kits (2 months old), which is consistent with studies on rats that report BALT first developing only after 2 weeks postnatally [46]. Although a general understanding of BALT may be extrapolated from related species, detailed studies in mink have not been published. Future research is necessary to investigate whether BALT plays a role in controlling respiratory infections and could be important for evaluation of environmental responses and farming practices.

The presence of lymphocytic aggregates in the lungs of healthy mink reported in this study warrants further investigation to determine any functional role. In addition, previous studies have suggested that mink could be used as a model for human COVID-19 studies [27, 42, 48] and the findings reported here emphasize the need for more detailed descriptions of mink lungs to evaluate suitability.

Furthermore, histological evidence for bacteria in the lungs of mink that died during SARS-CoV-2 outbreaks was minimal, suggesting that mortality is more likely due to damage caused by SARS-CoV-2. By contrast, secondary bacterial infections were reportedly responsible for mortality in Danish mink that succumbed to influenza infections [49].

In this study, proliferation of type II pneumocytes was predominantly observed in the group of mink in recovery from SARS-CoV-2, whereas the two groups of mink with active infections exhibited minimal proliferation in only a few animals. When assessing type II pneumocyte proliferation in mink lungs, AD status must be considered because the early stages of infection with AMDV can cause type II pneumocyte hyperplasia [50]. The mink included in this study were from farms that had been confirmed as free from AD before and after sampling. During the COVID-19 pandemic when SARS-CoV-2 outbreaks were occurring on mink farms worldwide, AD was widespread on mink farms globally [51]. In Denmark, AD is a notifiable disease and all mink breeders must test their animals several times every year. Strict testing regimes have successfully reduced outbreaks of AD on farms [52]. In a previous study conducted in the Netherlands, moderate to severe proliferation of type II pneumocytes was observed in adult mink from four different SARS-CoV-2-infected farms [22]. All these farms had tested positive for AMDV during the past 10 years, but none showed clinical signs of Aleutian disease. In another study from a SARS-CoV-2 farm outbreak in Utah, infrequent type II pneumocyte proliferation in 20 SARS-CoV-2 infected mink from an AD-positive farm was reported [11]. In two experimental studies of SARS-CoV-2 infected mink, type II pneumocyte proliferation was reported to moderate and varying degrees [42, 53], although the AD status of the mink was not specified. In another experimental study on 11 SARS-CoV-2 infected mink and 6 control mink, type II pneumocyte proliferation was not observed and, interestingly, these mink were AD negative [54]. The previous findings together with our observations, showing that proliferation predominately occurs 1 month after active SARS-CoV-2 infection, suggest that type II pneumocyte proliferation in SARS-CoV-2 infected mink may be a sign of post-infection recovery rather than active infection.

This study facilitated a comparative analysis of pulmonary histological lesions in SARS-CoV-2 infected mink kits with and without clinical disease, as well as mink in recovery from the disease. The inclusion of age matched controls was a marked strength of this study, given the lack of descriptions of normal mink pulmonary anatomy found in previous reports.

In all study groups, PCA were observed in the alveolar lumen of many animals. The presence of PCA in humans with pneumonia has been documented for decades [55–57], and PCA also occur in alveolar spaces in sheep with chronic and acute pneumonia [58, 59]. In general, PCA are considered incidental findings [60] and their specific function remains unknown [61], although their presence may indicate previous injury or pre-existing lesions wherein cellular debris and secretions have accumulated [56]. To our knowledge, this is the first study to document the presence of PCA in mink, and our observations suggest that the presence of PCA may be a common incidental finding in mink lungs.

## Conclusion

Severe pulmonary lesions were observed in SARS-CoV-2 infected mink kits with subclinical and clinical disease and in kits in recovery from infection. These lesions included epithelial damage, luminal secretion and hemorrhage, subepithelial inflammation of the bronchi and bronchioles, diffuse alveolar damage with hyaline membrane formation, alveolar hemorrhage, mononuclear cell infiltration of interstitia, and the formation of fibrin, edema, and thrombi. These findings suggest that the impact of SARS-CoV-2 farm outbreaks may be more serious than records of clinical signs indicate. The current surveillance system on Danish mink farms does not effectively detect repeated SARS-CoV-2 outbreaks that show no clinical signs or cause no mortality, which can lead to unnoticed welfare issues. The severity of the observed lesions reveal hidden health and welfare challenges in mink, underscoring the need for improved prevention measures, surveillance and understanding of long-term impact of SARS-CoV-2 infection in mink.

## Supplementary Information

Additional file 1. Semiquantitative scoring protocol for evaluating minklung tissue. Semiquantitative scoring protocol for histological evaluation of pulmonary tissue from farm mink infected with SARS-CoV-2. The protocol comprises six main categories with subcategories.

## Data Availability

The datasets used and/or analyzed during the current study are available from the corresponding author on reasonable request.

## Abbreviations

AD: Aleutian disease
AMDV: Aleutian mink disease virus
Con_July: Controls July
Con_Nov: Controls November
COVID-19: Coronavirus disease 2019
CS: Clinical SARS-CoV-2
IHC: Immunohistochemistry
PCA: Pulmonary corpora amylacea
RS: Recovering SARS-CoV-2
RT-qPCR: Reverse transcription-quantitative polymerase chain reaction
SARS-CoV-2: Severe acute respiratory syndrome coronavirus 2
SCS: Subclinical SARS-CoV-2

## References

[1] World Health Organization. Coronavirus disease (COVID-19)—situation reports [Internet]. 2024 [cited 2024 Nov 11]. https://www.who.int/emergencies/diseases/novel-coronavirus-2019/situation-reports

[2] Gortázar C, Barroso-Arévalo S, Ferreras-Colino E, Isla J, de la Fuente G, Rivera B, et al. Natural SARS-CoV-2 infection in kept ferrets, Spain. Emerg Infect Dis. 2021;27:1994–6. 34152974 10.3201/eid2707.210096 PMC8237878

[3] Hamer SA, Ghai RR, Zecca IB, Auckland LD, Roundy CM, Davila E, et al. SARS-CoV-2 B.1.1.7 variant of concern detected in a pet dog and cat after exposure to a person with COVID-19, USA. Transbound Emerg Dis. 2022;69:1656–8. 33955193 10.1111/tbed.14122 PMC8242881

[4] McAloose D, Laverack M, Wang L, Killian ML, Caserta LC, Yuan F, et al. From People to Panthera: Natural SARS-CoV-2 infection in tigers and lions at the Bronx Zoo. MBio. 2020;11:e02220. 33051368 10.1128/mBio.02220-20 PMC7554670

[5] World Organization for Animal Health. SARS-CoV-2 [Internet]. WOAH—World Organisation for Animal Health. 2024 [cited 2024 Nov 11]. https://www.woah.org/en/disease/sars-cov-2/

[6] Sailleau C, Dumarest M, Vanhomwegen J, Delaplace M, Caro V, Kwasiborski A, et al. First detection and genome sequencing of SARS-CoV-2 in an infected cat in France. Transbound Emerg Dis. 2020. 10.1111/tbed.13659. 32500944 10.1111/tbed.13659 PMC7300955

[7] Sit THC, Brackman CJ, Ip SM, Tam KWS, Law PYT, To EMW, et al. Infection of dogs with SARS-CoV-2. Nature. 2020;586:776–8. 32408337 10.1038/s41586-020-2334-5 PMC7606701

[8] Oreshkova N, Molenaar RJ, Vreman S, Harders F, Oude Munnink BB, Hakze-van der Honing RW, et al. SARS-CoV-2 infection in farmed minks, The Netherlands, April and May 2020. Euro Surveill. 2020. 10.2807/1560-7917.ES.2020.25.23.2001005. 32553059 10.2807/1560-7917.ES.2020.25.23.2001005 PMC7403642

[9] Larsen HD, Fonager J, Lomholt FK, Dalby T, Benedetti G, Kristensen B, et al. Preliminary report of an outbreak of SARS-CoV-2 in mink and mink farmers associated with community spread, Denmark, June to November 2020. Euro Surveill. 2021;26:2100009. 33541485 10.2807/1560-7917.ES.2021.26.5.210009 PMC7863232

[10] Hammer AS, Quaade ML, Rasmussen TB, Fonager J, Rasmussen M, Mundbjerg K, et al. SARS-CoV-2 transmission between mink (*Neovison** vison*) and humans. Denmark Emerg Infect Dis. 2021;27:547–51. 33207152 10.3201/eid2702.203794 PMC7853580

[11] Eckstrand CD, Baldwin TJ, Rood KA, Clayton MJ, Lott JK, Wolking RM, et al. An outbreak of SARS-CoV-2 with high mortality in mink (*Neovison** vison*) on multiple Utah farms. PLoS Pathog. 2021;17: e1009952. 34767598 10.1371/journal.ppat.1009952 PMC8589170

[12] European Centre for Disease Prevention and Control. Detection of new SARS-CoV-2 variants related to mink—12 November 2020. Stockholm: ECDC; 2020.

[13] Moreno A, Lelli D, Trogu T, Lavazza A, Barbieri I, Boniotti M, et al. SARS-CoV-2 in a mink farm in Italy: case description, molecular and serological diagnosis by comparing different tests. Viruses. 2022;14:1738. 36016360 10.3390/v14081738 PMC9415545

[14] Paiero A, Newhouse E, Chan E, Clair V, Russell S, Zlonsnik J, et al. SARS-CoV-2 in mink farms in British Columbia, Canada: a report of two outbreaks in 2020–2021. Can Commun Dis Rep. 2022;48:274–81. 37333572 10.14745/ccdr.v48i06a05 PMC10274534

[15] Domańska-Blicharz K, Orłowska A, Smreczak M, Niemczuk K, Iwan E, Bomba A, et al. Mink SARS-CoV-2 infection in Poland—short communication. J Vet Res. 2021;65:1–5. 33817389 10.2478/jvetres-2021-0017 PMC8009592

[16] Badiola JJ, Otero A, Sevilla E, Marín B, García Martínez M, Betancor M, et al. SARS-CoV-2 outbreak on a Spanish mink farm: epidemiological, molecular, and pathological studies. Front Vet Sci. 2022. 10.3389/fvets.2021.805004. 35127883 10.3389/fvets.2021.805004 PMC8814420

[17] Statistics Denmark. Fakta om minkbranchen i Danmark [Internet]. Danmarks stat. 2021 [cited 2024 Nov 19]. https://www.dst.dk/da/Statistik/nyheder-analyser-publ/bagtal/2020/2020-10-28-fakta-om-minkbranchen-i-Danmark

[18] Danish Veterinary and Food Administration. CHR [Internet]. 2024 [cited 2024 Nov 11]. https://chr.fvst.dk/chri/faces/frontpage

[19] Ministeriet for Fødevarer, Landbrug og Fiskeri. Bekendtgørelse om COVID-19 i pelsdyr. Nov 9, 2023.

[20] Boklund A, Hammer AS, Quaade ML, Rasmussen TB, Lohse L, Strandbygaard B, et al. SARS-CoV-2 in Danish mink farms: course of the epidemic and a descriptive analysis of the outbreaks in 2020. Animals (Basel). 2021;11:164. 33445704 10.3390/ani11010164 PMC7828158

[21] Chaintoutis SC, Thomou Z, Mouchtaropoulou E, Tsiolas G, Chassalevris T, Stylianaki I, et al. Outbreaks of SARS-CoV-2 in naturally infected mink farms: Impact, transmission dynamics, genetic patterns, and environmental contamination. PLoS Pathog. 2021;17: e1009883. 34492088 10.1371/journal.ppat.1009883 PMC8448373

[22] Molenaar RJ, Vreman S, Hakze-van der Honing RW, Zwart R, de Rond J, Weesendorp E, et al. Clinical and pathological findings in SARS-CoV-2 disease outbreaks in farmed mink (*Neovison** vison*). Vet Pathol. 2020;57:653–7. 32663073 10.1177/0300985820943535

[23] Feng A, Bevins S, Chandler J, DeLiberto TJ, Ghai R, Lantz K, et al. Transmission of SARS-CoV-2 in free-ranging white-tailed deer in the United States. Nat Commun. 2023;14:4078. 37429851 10.1038/s41467-023-39782-x PMC10333304

[24] Haagmans BL, Koopmans MPG. Spreading of SARS-CoV-2 from hamsters to humans. Lancet. 2022;399:1027–8. 35279247 10.1016/S0140-6736(22)00423-8 PMC8912938

[25] Oude Munnink BB, Sikkema RS, Nieuwenhuijse DF, Molenaar RJ, Munger E, Molenkamp R, et al. Transmission of SARS-CoV-2 on mink farms between humans and mink and back to humans. Science. 2021;371:172–7. 33172935 10.1126/science.abe5901 PMC7857398

[26] Fenollar F, Mediannikov O, Maurin M, Devaux C, Colson P, Levasseur A, et al. Mink, SARS-CoV-2, and the human-animal interface. Front Microbiol. 2021;12: 663815. 33868218 10.3389/fmicb.2021.663815 PMC8047314

[27] Ritter JM, Wilson TM, Gary JM, Seixas JN, Martines RB, Bhatnagar J, et al. Histopathology and localization of SARS-CoV-2 and its host cell entry receptor ACE2 in tissues from naturally infected US-farmed mink (*Neovison** vison*). Vet Pathol. 2022;59:681–95. 35229669 10.1177/03009858221079665

[28] Rasmussen TB, Fonager J, Jørgensen CS, Lassaunière R, Hammer AS, Quaade ML, et al. Infection, recovery and re-infection of farmed mink with SARS-CoV-2. PLoS Pathog. 2021;17: e1010068. 34780574 10.1371/journal.ppat.1010068 PMC8629378

[29] Jespersen A, Jensen H, Agger J, Heegaard P, Damborg P, Aalbaek B, et al. The effect of color type on early wound healing in farmed mink (*Neovison** vison*). BMC Vet Res. 2017. 10.1186/s12917-017-1052-1. 28532438 10.1186/s12917-017-1052-1 PMC5440898

[30] Jensen HE. Necropsy : a handbook and atlas. 1st ed. Frederiksberg: Biofolia; 2011.

[31] Hammer AS, Pedersen PE, Clausen T. Lærebog for “Sundhed og sygdom hos mink.” Biovet; 2022.

[32] Bancroft JD, Stevens A. Theory and practice of histological techniques. 4th ed. Edinburgh: Churchill Livingstone; 1996.

[33] Luna LG. Methods for cennective tissue—Masson’s Thrichrome method. Histologic staining methods of the Armed Forces Institute of Pathology. 3rd ed. New York: McGraw-Hill Book Company; 1968. p. 94–5.

[34] Corman VM, Landt O, Kaiser M, Molenkamp R, Meijer A, Chu DK, et al. Detection of 2019 novel coronavirus (2019-nCoV) by real-time RT-PCR. Euro Surveill. 2020;25:2000045. 31992387 10.2807/1560-7917.ES.2020.25.3.2000045 PMC6988269

[35] Rasmussen TB, Qvesel AG, Pedersen AG, Olesen AS, Fonager J, Rasmussen M, et al. Emergence and spread of SARS-CoV-2 variants from farmed mink to humans and back during the epidemic in Denmark, June–November 2020. PLos Pathog. 2020;20:e1012039. 10.1371/journal.ppat.1012039. 10.1371/journal.ppat.1012039 PMC11244769 38950065

[36] Larivière S. Mustela vison. Mamm Species. 1999. 10.2307/3504420.

[37] Horwitz DF, Rodan I. Behavioral awareness in the feline consultation: Understanding physical and emotional health. J Feline Med Surg. 2018;20:423. 29706091 10.1177/1098612X18771204 PMC11395291

[38] van Zeeland Y, Schoemaker N. Pain recognition in ferrets. Vet Clin North Am Exot Anim Pract. 2023;26:229–43. 36402483 10.1016/j.cvex.2022.07.011

[39] Henriksen BIF, Møller SH, Malmkvist J. Animal welfare measured at mink farms in Europe. Appl Anim Behav Sci. 2022;248: 105587.

[40] Lu L, Sikkema RS, Velkers FC, Nieuwenhuijse DF, Fischer EAJ, Meijer PA, et al. Adaptation, spread and transmission of SARS-CoV-2 in farmed minks and associated humans in The Netherlands. Nat Commun. 2021;12:6802. 34815406 10.1038/s41467-021-27096-9 PMC8611045

[41] Zhou J, Peacock TP, Brown JC, Goldhill DH, Elrefaey AME, Penrice-Randal R, et al. Mutations that adapt SARS-CoV-2 to mink or ferret do not increase fitness in the human airway. Cell Rep. 2022;38: 110344. 35093235 10.1016/j.celrep.2022.110344 PMC8768428

[42] Song Z, Bao L, Deng W, Liu J, Ren E, Lv Q, et al. Integrated histopathological, lipidomic, and metabolomic profiles reveal mink is a useful animal model to mimic the pathogenicity of severe COVID-19 patients. Sig Transduct Target Ther. 2022;7:1–13. 10.1038/s41392-022-00891-6 PMC8795751 35091528

[43] Hansen MS, Krog JS, Hjulsager CK, Chriel M, Larsen LE, Kokotovic B. Lungebetændelse hos mink med ansamlinger af mononucleære inflammationsceller. Annual report—Kopenhagen Fur. 2016. p. 113–9.

[44] Altikriti M, Khamas W, Henry RW. Light microscopy of bronchial associated lymphoid tissue of healthy domestic cat with suggested new nomenclature. Anat Physiol. 2012;2:1–4.

[45] Pabst R, Gehrke I. Is the bronchus-associated lymphoid tissue (BALT) an integral structure of the lung in normal mammals, including humans? Am J Respir Cell Mol Biol. 1990;3:131–5. 2378747 10.1165/ajrcmb/3.2.131

[46] Gregson RL, Davey MJ, Prentice DE. Postnatal development of bronchus-associated lymphoid tissue (Balt) in the rat *Rattus Norvegicus*. Lab Anim. 1979;13:231–8. 162237 10.1258/002367779780937870

[47] Plesch BEC, Gamelkoorn GJ, van de Ende M. Development of bronchus associated lymphoid tissue (BALT) in the rat, with special reference to T- and B-cells. Dev Comp Immunol. 1983;7:179–88. 6601592 10.1016/0145-305x(83)90066-6

[48] Shou S, Liu M, Yang Y, Kang N, Song Y, Tan D, et al. Animal models for COVID-19: hamsters, mouse, ferret, mink, tree shrew, and non-human primates. Front Microbiol. 2021. 10.3389/fmicb.2021.626553. 34531831 10.3389/fmicb.2021.626553 PMC8438334

[49] Chriél M, Jensen TH, Hjulsager C, Larsen LE, Jørgensen PH, Harslund JLF, et al. Consequences of outbreaks of influenza A virus in farmed mink (*Neovison vison*) in Denmark in 2009 and 2010. In: Larsen PF, Møller SH, Clausen T, Hammer AS, Lássen TM, Nielsen VH, et al., editors. Proceedings of the Xth International Scientific Congress in fur animal production. Wageningen: Academic Publishers; 2012. p. 186–9.

[50] Vahedi SM, SalekArdestani S, Banabazi MH, Clark F. Epidemiology, pathogenesis, and diagnosis of Aleutian disease caused by Aleutian mink disease virus: A literature review with a perspective of genomic breeding for disease control in American mink (*Neogale** vison*). Virus Res. 2023;336: 199208. 37633597 10.1016/j.virusres.2023.199208 PMC10474236

[51] Zaleska-Wawro M, Szczerba-Turek A, Szweda W, Siemionek J. Seroprevalence and molecular epidemiology of Aleutian Disease in various countries during 1972–2021: a review and meta-analysis. Animals. 2021;11:2975. 34679996 10.3390/ani11102975 PMC8533000

[52] Dam-Tuxen R, Dahl J, Jensen TH, Dam-Tuxen T, Struve T, Bruun L. Diagnosing Aleutian mink disease infection by a new fully automated ELISA or by counter current immunoelectrophoresis: a comparison of sensitivity and specificity. J Virol Methods. 2014;199:53–60. 24462658 10.1016/j.jviromet.2014.01.011

[53] Virtanen J, Aaltonen K, Kegler K, Venkat V, Niamsap T, Kareinen L, et al. Experimental infection of mink with SARS-COV-2 Omicron variant and subsequent clinical disease. Emerg Infect Dis. 2022;28:1286–8. 35608951 10.3201/eid2806.220328 PMC9155874

[54] Adney DR, Lovaglio J, Schulz JE, Yinda CK, Avanzato VA, Haddock E, et al. Severe acute respiratory disease in American mink experimentally infected with SARS-CoV-2. JCI Insight. 2022. 10.1172/jci.insight.159573. 36509288 10.1172/jci.insight.159573 PMC9746805

[55] Michaels L, Levene C. Pulmonary corpora amylacea. J Pathol Bacteriol. 1957;74:49–56.

[56] Hollander DH, Hutchins GM. Central spherules in pulmonary corpora amylacea. Arch Pathol Lab Med. 1978;102:629–30. 82433

[57] Dobashi M, Yuda F, Narabayashi M, Imai Y, Isoda N, Obata K, et al. Histopathological study of corpora amylacea pulmonum. Histol Histopathol. 1989;4:153–65. 2485191

[58] Azizi S, Korani FS, Oryan A. Pneumonia in slaughtered sheep in south-western Iran: pathological characteristics and aerobic bacterial aetiology. Vet Ital. 2013;49:109–18. 23564592

[59] Lin X, Alley MR, Manktelow BW, Slack P. Pulmonary corpora amylacea in sheep. J Comp Pathol. 1989;100:267–74. 2470790 10.1016/0021-9975(89)90104-7

[60] Abdelsalam EB, Al Sadrani AA. Incidental findings of pathological significance in pneumonic lungs of sheep in Al Qassim Area, Kingdom of Saudi Arabia: an abattoir survey. Comp Clin Pathol. 2015;24:951–5.

[61] Riba M, del Valle J, Augé E, Vilaplana J, Pelegrí C. From *corpora **amylacea* to wasteosomes: History and perspectives. Ageing Res Rev. 2021;72: 101484. 34634491 10.1016/j.arr.2021.101484

