# Supplementary material for: Subclinical and long-term effects of severe acute respiratory syndrome coronavirus 2 infection in Danish farmed mink: implications for disease surveillance
Source: Acta Vet Scand. 2025 Jun 2;67:29. doi: 10.1186/s13028-025-00813-w (PMC12131573; doi:10.1186/s13028-025-00813-w)
Supplement: Supplementary file 1 — Additional file 1. Semiquantitative scoring protocol for evaluating minklung tissue. Semiquantitative scoring protocol for histological evaluation of pulmonary tissue from farm mink infected with SARS-CoV-2. The protocol comprises six main categories with subcategories. [file 13028_2025_813_MOESM1_ESM.docx]

**Additional file 1: Semiquantitative scoring protocol for evaluating mink (*Neogale vison*) lung tissue**

Semiquantitative scoring protocol for histological evaluation of pulmonary tissue from farm mink infected with SARS-CoV-2. The protocol comprises six main categories with subcategories.

| **Bronchi** | | | | |
| --- | --- | --- | --- | --- |
| *Damage to epithelium* | | | | |
| 0 | Intact pseudostratified kinociliated columnar cells with goblet cells and serous cells. | | | |
| 1 | Mild: Partial or complete loss of cilia but intact epithelium. | | | |
| 2 | Moderate: Loss of cilia, degeneration, and desquamated epithelium. | | | |
| 3 | Severe: Degeneration and mucosal ulcerations. Basement membrane exposed. | | | |
| *Luminal secretion* | | | | |
| 0 | Clear lumen and thin layer of secretion. | | | |
| 1 | Intraluminal secretion. | | | |
| 2 | Intraluminal secretion and debris. | | | |
| 3 | Intraluminal secretion, debris, and leukocytes. | | | |
| *Luminal hemorrhage* | | | | |
| 0 | <5 erythrocytes in the lumen. | | | |
| 1 | 5–10 erythrocytes in the lumen. | | | |
| 2 | 11–20 erythrocytes in the lumen. | | | |
| 3 | More than 20 erythrocytes in the lumen. | | | |
| *Submucosal inflammation* | | | | |
| 0 | No submucosal inflammation. | | | |
| 1 | Mild: Focal submucosal inflammation. | | | |
| 2 | Moderate: Multifocal submucosal inflammation. | | | |
| 3 | Severe: Leucocyte cuffing/diffuse submucosal inflammation. | | | |
| **Glands** | | | | |
| *Inflammation* | | | | |
| 0 | No signs of inflammation. A maximum of leucocytes in two glands is acceptable. | | | |
| 1 | Mild: Up to one-third of the glands are inflamed. | | | |
| 2 | Moderate: Up to two-thirds of the glands are inflamed. | | | |
| 3 | Severe: More than two-thirds of the glands are inflamed. | | | |
| *Degeneration* | | | | |
| 0 | No signs of necrosis. A maximum of two glands with degenerative cells is acceptable. | | | |
| 1 | Mild: Up to one-third of the glands are necrotic. | | | |
| 2 | Moderate: Up to two-thirds of the glands are necrotic. | | | |
| 3 | Severe: More than two-thirds of the glands are necrotic. | | | |
| **Bronchioles** | | | | |
| *Damage to epithelium* | | | | |
| 0 | Intact epithelium: Either simple columnar or simple cuboidal. Ciliated cells are present. | | | |
| 1 | Mild: Loss of cilia but intact epithelium and/or less than five degenerate cells. | | | |
| 2 | Moderate: Degeneration and/or desquamated epithelium. | | | |
| 3 | Severe: Mucosal ulcerations. Basement membrane exposed. | | | |
| *Luminal secretion* | | | | |
| 0 | Clear lumen and thin layer of secretion. | | | |
| 1 | Intraluminal secretion. | | | |
| 2 | Intraluminal secretion and debris. | | | |
| 3 | Intraluminal secretion, debris, and leukocytes. | | | |
| *Luminal hemorrhage* | | | | |
| 0 | <5 erythrocytes in the lumen. | | | |
| 1 | 5–10 erythrocytes in the lumen. | | | |
| 2 | 11–20 erythrocytes in the lumen. | | | |
| 3 | More than 20 erythrocytes in the lumen. | | | |
| *Submucosal inflammation* | | | | |
| 0 | No submucosal inflammation. | | | |
| 1 | Mild: Focal submucosal inflammation. | | | |
| 2 | Moderate: Multifocal submucosal inflammation. | | | |
| 3 | Severe: Leucocyte cuffing/diffuse submucosal inflammation. | | | |
|  |  | | | |
| **Vascular system** | | | | |
| *Distribution of vascular lesions* | | | | |
| 0 | No vascular lesions. | | | |
| 1 | Focal vascular lesions. | | | |
| 2 | Multifocal vascular lesions. | | | |
| 3 | Diffuse vascular lesions. | | | |
| *Vasculitis* | | | | |
| 0 | No signs of vasculitis. | | | |
| 1 | Mild: Up to one-third of the vessels. | | | |
| 2 | Moderate: Up to two-thirds of the vessels. | | | |
| 3 | Severe: More than two-thirds of the vessels. | | | |
| *Perivasculitis* | | | | |
| 0 | No signs of perivasculitis. | | | |
| 1 | Up to one-third of the vessels. | | | |
| 2 | Up to two-thirds of the vessels. | | | |
| 3 | More than two-thirds of the vessels. | | | |
| *Width of perivasculitis* | | | | |
| 0 | None. | | | |
| 1 | Mild: Two layers of inflammatory cells. | | | |
| 2 | Moderate: Three or four layers of inflammatory cells. | | | |
| 3 | Severe: More than four layers of inflammatory cells. | | | |
| *Perivascular edema* | | | | |
| 0 | No signs of perivascular edema. | | | |
| 1 | Up to one-third of the vessels. | | | |
| 2 | Up to two-thirds of the vessels. | | | |
| 3 | More than two-thirds of the vessels. | | | |
| *Width of perivascular edema* | | | | |
| 0 | None | | | |
| 1 | Mild: Local edema around the vessel, corresponding to one layer of mononuclear cells. | | | |
| 2 | Moderate: Edema corresponding to two to four layers of mononuclear cells. | | | |
| 3 | Severe: Edema corresponding to more than four layers of inflammatory cells. | | | |
| *Perivascular accumulation of mononuclear cells* | | | | |
| Mononuclear cells observed without the presence of vasculitis, edema, or other signs of an inflammatory response. | | | | |
| 0 | None. | | | |
| 1 | Mild: Two layers of mononuclear cells. | | | |
| 2 | Moderate: Three to four layers of mononuclear cells. | | | |
| 3 | Severe: More than four layers of mononuclear cells. | | | |
|  |  | | | |
| **Alveolar septal interstitium** | | | | |
| *Cell infiltration in the interstitium* | | | | |
| 0 | No infiltration (one macrophage, lymphocyte, or neutrophil per high-powered field [400× magnification] is considered normal). | | | |
| 1 | Focal infiltration. | | | |
| 2 | Multifocal infiltration. | | | |
| 3 | Diffuse infiltration. | | | |
| *Type II pneumocyte proliferation* | | | | |
| 0 | No proliferation observed. | | | |
| 1 | Local proliferation. | | | |
| 2 | Multifocal proliferation. | | | |
| 3 | Diffuse proliferation. | | | |
| *Alveolar damage* | | | | |
| 0 | No alveolar damage. | | | |
| 1 | Mild: Congestion, mild edema, and alveolar hemorrhage. Few hyaline membranes. | | | |
| 2 | Moderate: Congestion, extensive edema with multiple hyaline membranes. Hemorrhage in more than half of the alveolar lumen. Partial atelectasis. | | | |
| 3 | Severe: Congestion, extensive edema with multiple hyaline membranes. Hemorrhage in more than half of the alveolar lumen. Severe atelectasis. | | | |
|  |  | | | |
| **Alveolar lumen** | | | | |
| *Alveolar inflammation* | | | | |
| 0 | No inflammation. The presence of two alveolar macrophages is considered normal. | | | |
| 1 | Focal inflammation. | | | |
| 2 | Multifocal inflammation. | | | |
| 3 | Diffuse inflammation. | | | |
| *Alveolar fibrin* | | | | |
| 0 | No fibrin in the lumen. | | | |
| 1 | Focal. | | | |
| 2 | Multifocal. | | | |
| 3 | Diffuse. | | | |
| *Alveolar hemorrhage* | | | | |
| 0 | No alveolar hemorrhage. | | | |
| 1 | Focal. | | | |
| 2 | Multifocal. | | | |
| 3 | Diffuse. | | | |
| *Luminal edema* | | | | |
| 0 | No edema. | | | |
| 1 | Focal. | | | |
| 2 | Multifocal. | | | |
| 3 | Diffuse. | | | |
| *Reduction of alveolar airspace* | | | | |
| Evaluated at 25× magnification | | | | |
| 0 | Normal alveolar space. | | | |
| 1 | Mild: Up to one-third reduced. | | | |
| 2 | Moderate: Up to two-thirds reduced. | | | |
| 3 | Severe: More than two-thirds reduced. | | | |
|  |  | | | |
|  |  | | | |
|  |  | | | |
| **Dichotomous scale** | | | | |
| Intracellular granules in macrophages | | | Present [ ] | Not present [ ] |
| Giant cells | | | Present [ ] | Not present [ ] |
| Bacteria | | | Present [ ] | Not present [ ] |
| Thrombi | | | Present [ ] | Not present [ ] |
| Neutrophils in the alveolar lumen or septae | | | Present [ ] | Not present [ ] |
| Pulmonary corpora amylacea (PCA) | | | Present [ ] | Not present [ ] |
